# HLA-A, -B, -C, -DPB1, -DQB1 and -DRB1 allele frequencies of North Tanzanian Maasai

**DOI:** 10.1016/j.humimm.2022.10.008

**Published:** 2022-11-03

**Authors:** Amber Barton, Athumani Ramadhani, Elias Mafuru, Tara Mtuy, Patrick Massae, Aiweda Malissa, Tamsyn Derrick, Joanna Houghton, Anna Harte, Thomas Payne, Harry Pickering, Matthew J Burton, Chrissy H. Roberts, Martin J. Holland

**Affiliations:** a Clinical Research Department, Faculty of Infectious and Tropiclal Diseases, London School of Hygiene and Tropical Medicine, London, UK; b Eye Health Project, Kilimanjaro Christian Medical Centre, Moshi, Tanzania

**Keywords:** HLA, MHC, Tanzania, Maasai, Immunogenetics

## Abstract

Locus-specific amplicon sequencing was used to HLA type 336 participants of Maasai ethnicity at the HLA-A, -B, -C, -DRB1, -DQB1 and -DPB1 loci. Participants were recruited from three study villages in North Tanzania, for the purpose of investigating risk factors for trachomatous scarring in children. Other than HLA-A, all loci significantly deviated from Hardy-Weinberg equilibrium, possibly due to high relatedness between individuals: 238 individuals shared a house with at least one another participant. The most frequent allele for each locus were A*68:02 (14.3 %), B*53:01 (8.4 %), C*06:02 (19.2 %), DRB1*13:02 (17.7 %), DQB1*02:01 (16.9 %) and DPB1*01:01 (15.7 %), while the most common inferred haplotype was A*68:02 *~* B*18:01 *~* C*07:04 *~* DRB1*08:04 *~* DQB1*04:02 *~* DPB1*04:01 (1.3 %).

Children aged 6–10 (N = 336) were recruited from three neighbouring villages located between Mount Meru and Mount Kilimanjaro in Tanzania. Participants were predominantly of Maasai ethnicity. The Maasai, a Nilotic ethnic group, are thought to have originated from southern Sudan, migrating South to settle in North Tanzania and Kenya in the first millennium [3]. The Maasai have maintained a semi-nomadic pastoralist lifestyle and Nilo-Saharan language, Maa. Villages are organised into bomas: joint residential units with one or more families in houses encircling an animal enclosure [4]. Polygyny is common [2]. The study villages were remote with similar lifestyles, and were selected on the basis of having an active trachoma prevalence greater than 30 % in children [5]. Children aged 6–10 were selected as they were most likely to show progressive scarring over the course of the longitudinal study. 238 individuals shared a house with at least one another participant. This study was reviewed and approved by the Ethics Committees of the Tanzania National Institute for Medical Research, Kilimanjaro Christian Medical University College and the London School of Hygiene & Tropical Medicine. Field workers explained the study to parents in Swahili or Maa language, and children were enrolled if parents gave written consent.

Human leukocyte antigen (HLA)-A, -B, -C, -DRB1, -DQB1 and -DPB1 were typed to four-digit resolution by DNA amplicon sequencing, Briefly, DNA was isolated from ocular dry swabs using a QIAamp DNA mini kit (Qiagen). HLA-A, -B, -C, -DRB1, -DQB1 and -DPB1 amplicons were generated by locus-specific PCR amplification (32 cycles for class I loci, 30 cycles for class II loci) then run on an agarose gel to check for amplicons of the correct size. Amplicons were pooled for each participant then underwent six cycles of indexing PCR. Groups of 16 samples were pooled and cleaned using 0.7 v/v AMPure XP beads (Beckman Coulter). Pools were diluted to 12.5 nM then combined. A final sample library at concentration 13.5 pM with 1.25 pM control phiX library spiked in was loaded into a MiSeq flow cell. 2 × 292 base-pair paired end sequencing was carried out using the MiSeq Next Generation Sequencing Platform. Seqtk was used to sub-sample 15,000 reads from fastq files, using the same random seed to ensure reads remained paired. Burrows-Wheeler Aligner was used to align reads to chromosome 6, and SAMtools to convert sequence data to binary bam format, then sort and index reads. Samples were then HLA typed using xHLA [6,7], which aligns sequenced reads to the IGMT/HLA database using protein-level aligner DIAMOND, and accepts only perfect matches.

As only perfect matches were accepted, 335, 334, 320, 327, 335 and 331 participants were successfully typed at the HLA-A, -B, -C, -DRB1, -DQB1 and -DPB1 loci respectively. While it is possible this missingness introduced bias to the allele frequencies, 313/336 (93 %) of participants had no missing loci. Allele frequencies were calculated by direct counting in the R statistical environment (Supplemental Table I). The most frequent alleles at each locus were A*68:02 (14.3 %), B*53:01 (8.4 %), C*06:02 (19.2 %), DRB1*13:02 (17.7 %), DQB1*02:01 (16.9 %) and DPB1*01:01 (15.7 %). 38, 53, 78, 26, 17 and 30 unique alleles were detected at the HLA-A, -B, -C, -DRB1, -DQB1 and -DPB1 loci respectively. However, certain alleles are likely over- or under- represented relative to the general Maasai population due to relatedness within the villages. Whereas the median number of alleles shared between participants from different bomas was 1/12 (interquartile range (IQR) 1–2), participants in the same boma shared a median of 2/12 alleles (IQR 1–3) and participants in the same house 7/12 (IQR 2–12). Considering that 238 individuals shared a house with at least one another participant, there was therefore a considerable degree of relatedness. Allele frequencies for a random subset of one participant per boma are shown in Supplemental Table II, with the caveat of a much smaller sample size (n = 70).

Arlequin version 3.5.22 was used to infer haplotypes using an expectation–maximisation algorithm and calculate deviation from Hardy-Weinberg equilibrium. This analysis was limited to participants with no missing loci. Of 12,297 possible haplotypes, 458 had an estimated frequency above 1 × 10^−5^ (Supplemental Table III). Three haplotypes had an estimated frequency above 1 %, the most frequent of which was A*68:02 *~* B*18:01 *~* C*07:04 *~* DPB1*04:01 *~* DQ B1*04:02 *~* DRB1*08:04 (1.3 %). All loci significantly deviated from Hardy-Weinberg equilibrium (p < 0.05) apart from HLA-A (p = 0.07), with lower than expected heterozygotes (Supplemental Table IV). Sub-setting participants to a random sample of one participant per boma brought the HLA-DPB1 and -DQB1 loci into Hardy-Weinberg equilibrium (n = 70 genotypes, p = 0.55, 0.0018, 0.00079, 0.27, 0.41, and 0.054 for HLA-A, -B, -C, -DPB1, -DQB1 and -DPB1 respectively). This suggests that unaccounted for relatedness between individuals, which inflates type I error [1], likely has an effect on the observed genotype frequencies. The results reported here therefore likely do not capture the full diversity of HLA alleles in the Maasai population. Population sub-structure or genotyping error could also play a role. The data presented here is deposited in the Allele Frequencies Net Database under identifier 3782, “Tanzania Maasai”.

## Supplementary Material

Supplementary data to this article can be found online at https://doi.org/10.1016/j.humimm.2022.10.008.

1

2

3

4
